# Minireview: Peripheral Nesfatin-1 in Regulation of the Gut Activity—15 Years since the Discovery

**DOI:** 10.3390/ani12010101

**Published:** 2022-01-01

**Authors:** Katarzyna Kras, Siemowit Muszyński, Ewa Tomaszewska, Marcin B. Arciszewski

**Affiliations:** 1 Department of Animal Anatomy and Histology, Faculty of Veterinary Medicine, University of Life Sciences in Lublin, 12 Akademicka St., 20-950 Lublin, Poland; katarzyna.kras@up.lublin.pl; 2 Department of Biophysics, Faculty of Environmental Biology, University of Life Sciences in Lublin, 13 Akademicka St., 20-950 Lublin, Poland; siemowit.muszynski@up.lublin.pl; 3 Department of Animal Physiology, Faculty of Veterinary Medicine, University of Life Sciences in Lublin, 12 Akademicka St., 20-950 Lublin, Poland; ewaRST@interia.pl

**Keywords:** nesfatin-1, gut, gastrointestinal disorders, enteric neurons, gut microbiota, gut peptides

## Abstract

**Simple Summary:**

Nesfatin-1 is a newly identified molecule derived from the precursor protein NEFA/nucleobindin2. In this minireview we analyzed the research on the nesfatin-1 localization in the gastrointestinal tract of the mammals. We also referred to the effects of the protein on disorders in the gastrointestinal tract.

**Abstract:**

Nesfatin-1, discovered in 2006, is an anorexigenic molecule derived from the precursor protein NEFA/nucleobindin2. It is generally postulated that this molecule acts through a specific G protein-coupled receptor, as yet unidentified. Research conducted over the last 15 years has revealed both central and peripheral actions of nesfatin-1. Given its major central role, studies determining its inhibitory effect on food intake seem to be of major scientific interest. However, in recent years a number of experiments have found that peripheral organs, including those of the gastrointestinal tract (GIT), may also be a source (possibly even the predominant source) of nesfatin-1. This mini-review aimed to summarize the current state of knowledge regarding the expression and immunoreactivity of nesfatin-1 and its possible involvement (both physiological and pathological) in the mammalian GIT. Research thus far has shown very promising abilities of nesfatin-1 to restore the balance between pro-oxidants and antioxidants, to interplay with the gut microbiota, and to alter the structure of the intestinal barrier. This necessitates more extensive research on the peripheral actions of this molecule. More in-depth knowledge of such mechanisms (especially those leading to anti-inflammatory and anti-apoptotic effects) is important for a better understanding of the involvement of nefatin-1 in GIT pathophysiological conditions and/or for future therapeutic approaches.

## 1. Introduction

**Figure 1:**
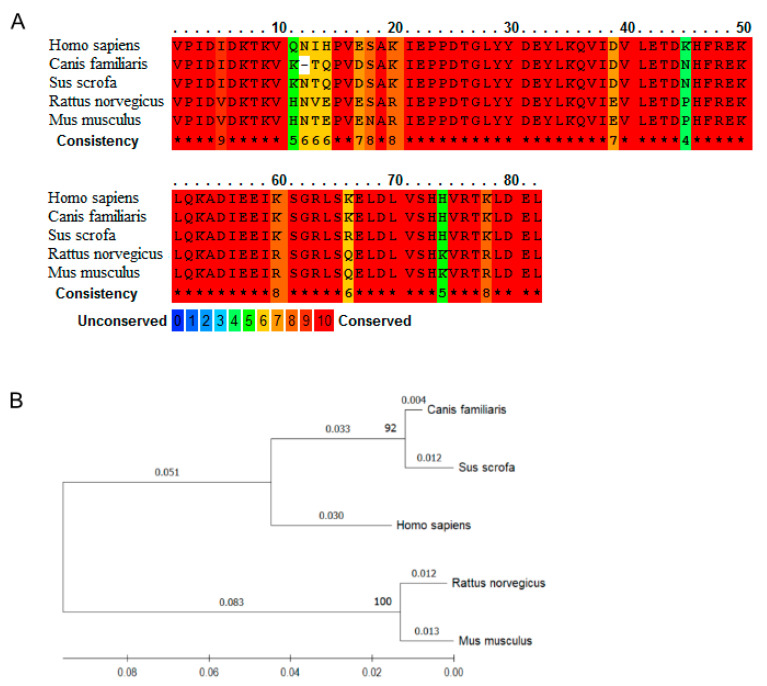
Comparison of nesfatin-1 sequence across (*Homo sapiens*), dog (*Canis familiaris*), pig (*Sus scrofa*), rat (*Rattus norvegicus*), and mouse (*Mus musculus*) shows a high homology at the amino acid level. (**A**) Nesfatin-1 amino acid sequence in human (UniProtKB: P80303), dog (UniProtKB: P80303), pig (UniProtKB: A0A5G2RI19), rat (UniProtKB: Q9JI85), and mouse (UniProtKB: P81117) were compared using the multiple sequence alignment program PRALINE [4] (https://www.ibi.vu.nl/programs/pralinewww, accessed on 13 December 2021). The scoring scheme works from 0 for the least conserved alignment position, up to 10 for the fully conserved alignment position (*). (**B**) Phylogenetic tree generated by the Maximum Likelihood method using the with Mega-X software (ver. 11.0.10) [5] based on amino acid sequences. The branch lengths reflect the degree of divergence of each sequence, numbers in bold on branches represent the bootstrap value.

The history of nesfatin-1 begins in 2006 when a group of researchers led by Shinsuke Oh-I identified a new anorexigenic molecule in the hypothalamus of the rat [1]. In a series of experiments involving immunohistochemistry (IHC) and in situ hybridization (ISH) techniques, they located a new protein encoded in the precursor protein NEFA/nucleobindin2, in the arcuate nucleus, paraventricular nucleus (PVN), supraoptic nucleus, lateral hypothalamic area, zona incerta, and nucleus tractus solitarius (NTS), which are believed to be hypothalamic nuclei involved in appetite regulation. The name ‘nesfatin’, i.e., NEFA/nucleobindin2-encoded satiety- and fat-influencing protein, is derived from its first noted activity. The peptide precursor, 396 aa in length, requires endoproteolytic postranslational processing to generate smaller biologically active fragments: nesfatin-1 (residues 1–82), nesfatin-2 (residues 85–163), and nesfatin-3 (residues 166–396). Among those three synthetic peptides only nesfatin-1 appeared able to decrease food intake, whereas nesfatin-2 and nesfatin-3 showed neither stimulatory nor inhibitory effects on appetite. One midsegment of nesfatin-1 (30 aa in length) seems to be of special importance, as its intraperitoneal injection resulted in activation of NTS neurons expressing c-fos [2]. During 15 years of research, the significance of nesfatin-1 was established, as it was shown to play both central and peripheral roles in numerous physiological and pathological processes. It must be noted, however, that most nesfatin-1 research has focused on its central action, while its peripheral role has attracted less attention. Several studies have shown that the primary source of nesfatin-1 might be not the central nervous system, but the peripheral organs of the gastrointestinal tract (GIT), predominantly the gastric mucosal endocrine cells [3]. For this reason, the activity of nesfatin-1 in the brain–gut axis and GIT controlling the enteric nervous system (ENS) is of particular importance. This minireview aims to summarize current knowledge on the possible interactions between peripherally secreted nesfatin-1 and the mammalian gut available in literature: human, dog, pig, rat, and mouse (Figure 1).

## 2. Mechanism of Action

Before characterizing the significance of nesfatin-1, the review will outline the current state of knowledge about possible mechanisms of its action. Despite some attempts, no specific intra- and/or extracellular receptor binding the peptide ligand has been identified, cloned, or characterized. However, there are several indications that nesfatin-1 acts through a specific receptor. First, autoradiography using ^125^I-nesfatin-1 has been used to detect specific binding sites for nesfatin-1 in the brain and numerous peripheral organs, including the stomach, duodenum, jejunum, ileum, and pancreas [6]. Second, nesfatin-1 was shown to stimulate Ca^+2^ influx in cultured hypothalamic neurons, and this elevation was significantly reduced by specific protein kinase A inhibitor KT 5720, which indicates an interaction between nesfatin-1 and G protein-coupled receptor [7]. It is noteworthy that, as shown in cultured dorsal root ganglia neurons, the elevated Ca^+2^ levels resulting from nesfatin-1 activity may also be achieved through a protein kinase C-dependent mechanism [8]. However, it must be kept in mind that nesfatin-1 may also act as a regulator of ion channels. Nesfatin-1 was shown to inhibit voltage-gated K^+^ (Kv) channel current of pancreatic mouse primary beta cells and to bind to Kv2.1 channels [9].

It has been established that nefatin-1 can be secreted and stored in a variety of cell types, and the distinctive immunoexpression patterns of nesfatin-1 throughout the cells may reflect its activity. Since no precise data describing ultrastructural nesfatin-1 distribution in peripheral nervous cells are available, some analogies to the sites of nesfatin-1 secretion and release in central neurons controlling the gut may be of importance. The use of specific antibodies directed against nesfatin-1 (but also against nucleobindin-2, NUCB2) has revealed an immune reaction in the cytoplasm (but not in the varicosities or nerve terminals) of neurons present in ventral sites of the forebrain, hypothalamus, and spinal cord [10,11]. Electron microscopy confirmed that nesfatin-1 is located in the secretory vesicles of PVN neurons and when released is able to activate the adjacent oxytocin-IR nervous cells, which strongly supports the hypothesis of a paracrine/autocrine mode of action [12]. Additionally, based on the example of the testis, it has been suggested that in non-neuronal peripheral tissues nesfatin-1 may act in a paracrine manner [13]. It is noteworthy that in the cells of peripheral organs, nesfatin-1 is usually visualized as granules exclusively limited to the cytoplasm and not present in the nucleus or cell membrane [14].

## 3. Expression and Immunolocalization of Nesfatin-1 in the Gut

The discovery that the brain–blood or blood–brain barrier is permeable to nesfatin-1 in a non-saturable manner [15] led to the suggestion that binding sites for the molecule might be present at the periphery. Given that cells of certain peripheral organs (mainly the stomach) are also a major source of secreted nesfatin-1, it is likely that the peripheral action of this peptide is more complex than its central action. In the last 15 years numerous studies utilizing a variety of techniques have focused on locating nesfatin-1 in the mammalian GIT (see Table 1 for summary).

Although Western blot (WB) analysis revealed that the rat esophagus is a minor source of nesfatin-1/NUCB2 compared to other segments of the digestive tract (nearly twofold lower expression in relation to the stomach), classic IHC failed to precisely locate the target protein within any cells [3,14]. The qRT-PCR technique was used to locate the expression of *NUCB2* mRNA in the esophagus of dogs in four different developmental stages (again the IHC was not useful for detecting IR cells), and interestingly, higher levels were found in juvenile animals [16]. One explanation for these phenomena may be that at least part of the gastroesophageal junction, which according to some authors is lined with a cardiac-like mucosa [17], was unintentionally removed together with the esophagus during dissection. Indeed, according to the previously mentioned WB analysis, the stomach seems to be the primary source of nesfatin-1 in the mammalian gut. Numerous studies in rodents, pigs, and humans have confirmed that the protein is mainly present in specific subsets of endocrine (but not parietal) cells of gastric oxyntic mucosa (at even higher levels than in the brain or heart) at all stages of development, although certain species-specific differences in the distribution pattern have been noted [3,14,18,19,20]. Interestingly, Stengel et al. [3] showed that nearly half of nesfatin-1-expressing endocrine cells also co-store ghrelin, and the vast majority of ghrelin-IR cells co-express nesfatin-1, which strongly indicates the functional cooperation of these two peptides. Minor populations of nesfatin-1 IR cells producing somatostatin or histidine decarboxylase were also identified. A more recent study clearly demonstrated that the rate of production of nesfatin-1 in the stomach of rodents is also age-dependent (higher in the fetus and lower in older animals) and probably reflects the need to adapt to different energy requirements [21,22]. 

Reports describing the presence of nesfatin-1/NUCB2 in the mammalian small intestine are sometimes contradictory. Higher expression of *NUCB2* mRNA was found in the canine duodenum than in the jejunum, and interestingly, these levels were higher in young animals than in adults [16]. Although the authors of that study were not able to identify any nesfatin-1-positive cells by immunohistochemical staining, Gonkowski et al. [23] used IHC methods to visualize a relatively numerous population of nesfatin-1-expressing cells limited to the surface epithelium (close to the enterocytes) of the canine small intestine. The presence of nesfatin-1/NUCB2 was also confirmed by WB analysis in the rat duodenum, with a strong indication that most of the protein was located in Brunner’s gland cells [14]. On the other hand, another interesting contribution clearly documented that nesfatin-1 in the rat small intestine (duodenum and jejunum) is mainly expressed in the Paneth cells within the crypts [24]. In contrast, Stengel et al. [3] reported that the small intestine of the adult rat contained no nesfatin-1-IR cells. Interestingly, in another experiment conducted on neonatal rats (13 and 27 days old), nesfatin-1/NUCB2 was found in the duodenal enteroendocrine cells [19]. It has been suggested that the distribution pattern of intestinal nesfatin-1 expression may be species-specific, since immunopositive endocrine cells were found in the ileocecal valve of adult Casertana pigs [20]. The most intriguing finding of that study is that immunoreactivity to nesfatin-1 was detected in both the myenteric and submucosal plexuses of the duodenum (predominantly the inner submucosal plexus) and ileum (predominantly the outer submucosal plexus). It is noteworthy that the presence of nesfatin-1 has also recently been shown in both enteric plexuses (myenteric and submucosal) innervating the duodenum in normal and gastrectomized rats [24]. The functional significance of these findings is far from fully understood. 

Data describing the expression patterns of nesfatin-1 in the large intestine are fragmentary. Primary studies utilizing the qRT-PCR and WB techniques revealed fairly low levels of *NUCB2* mRNA and nesfatin-1 protein in the large intestine of dogs, with certain differences depending on the age and segment [16]. A similar pattern was observed in the colon of rats and mice [14]. In Casertana pigs, low protein expression was detected in the cecum and colon, and no presence of *NUBC2* mRNA was found in the rectum [20]. While the results of WB studies are fairly consistent, the results of IHC analyses are quite divergent and sometimes contradictory. Immunohistochemical studies of the canine large intestine have revealed either a small population of nesfatin-1-IR colonic mucosal cells [23] or no presence of cells expressing nesfatin-1 [16]. Similarly to the latter case, the large intestine of rats and mice contained no cells immunopositive for nesfatin-1 [14]. On contrary, in pigs the presence of nefatin-1 was confirmed not only in colonic endocrine cells but also in myenteric (but not submucosal) neurons and nerve fibers [20].

**Table 1:** The localization and level of expression of the nesfatin-1 protein in the mammalian gastrointestinal tract.

	Species Studied	Method of Detection	Expression Level/Cellular Location	Adapted From
**Esophagus**	Rat/mouse	WB	+	[14]
	Rat	IHC	-	[3]
	Dog	IHC	-	[16]
**Stomach**	Rat/mouse	WB	+++	[14]
	Rat/mouse	IHC	Gastric mucosal glands	[14]
	Rat	WB	+	[3]
	Rat	IHC	Gastric oxyntic mucosa	[3]
	Dog	IHC	Fundic glands	[16]
	Human	IHC	Mucosa (cytoplasmic vesicle)	[18]
	Human	WB	+	[18]
	Rat	IHC	Oxyntic glands	[19]
	Pig	IHC	Fundic glands	[20]
	Dog	IHC	Mucosa	[23]
**Small intestine**	Rat	IHC	-	[3]
Duodenum	Rat/mouse	WB	+++	[14]
	Rat/mouse	IHC	Submucosa, Brunner’s glands	[14]
	Dog	IHC	-	[16]
	Rat	IHC	Enteroendocrine cells in the villi	[19]
	Pig	IHC	Neurons and nervous fibers in submucous and myenteric plexuses	[20]
	Dog	IHC	Mucosa	[23]
	Rat	IHC	Paneth cells, submucous and myenteric plexuses	[24]
Jejunum	Dog	IHC	-	[16]
	Pig	IHC	Neurons and nervous fibers in submucous and myenteric plexuses	[20]
	Dog	IHC	Mucosa	[23]
	Rat	IHC	Paneth cells, enterocytes, submucous and myenteric plexuses	[24]
Ileum	Dog	IHC	-	[16]
	Pig	IHC	Neurons and nervous fibers in submucous and myenteric plexuses	[20]
Ileocecal valve	Pig	IHC	Glands, submucous and myenteric plexuses	[20]
**Large intestine**				
Cecum	Dog	IHC	-	[16]
	Pig	IHC	Neurons and nervous fibers in submucous and myenteric plexuses	[20]
Colon	Rat/mouse	WB	+	[14]
	Rat	IHC	-	[3]
	Dog	IHC	-	[16]
	Pig	IHC	Neuronal cells in myenteric plexus	[20]
	Dog	IHC	Mucosa	[23]
Rectum	Dog	IHC	-	[16]
	Pig	IHC	-	[20]

IHC—Immunohistochemistry; WB—Western blot; The semiquantitative scale used: - absent, + low, +++ numerous.

## 4. Role of Nesaftin-1 in Gastrointestinal Tract Disorders

After 15 years of study, apart from its obvious anorexigenic central effect, the exact role of nesfatin-1 secreted peripherally from GIT cells is far from completely understood. In a rat model of ulceritis (induced by administration of indomethacin subcutaneously or a low level of acetic acid by gavage), nesfatin-1 injected intraperitoneally or intravenously substantially reduced epithelial desquamation of the gastric mucosa, neutrophil infiltration, erosion, and bleeding scores, and subsequently inhibited the generation of pro-inflammatory myeloperoxidase, malondialdehyde, chemiluminescence, tumor necrosis factor α (dose-dependently), and interleukin-1 [25,26]. In another model of rat chronic gastric ulcers (induced by serosal application of concentrated acetic acid), intraperitoneal administration of nesfatin-1 for 10 days substantially improved mucosa regeneration and healing; this protective effect was mediated by nitric oxide or by several sensory neuropeptides [27]. Taken together, these findings suggest that ulcer-healing nesfatin-1 may also be locally secreted, and the entire process may be at least partially controlled by the ENS, which is well known as a source of sensory and nitrergic neurons. It is noteworthy that rats subjected to gastric vagotomy (as a result of a Roux-en-Y gastric bypass) showed increased expression of nesfatin-1 in the stomach as well as increased postprandial nesfatin-1 levels in the portal vein [28]. In that experiment, the overriding control of the gut by the central nervous system was virtually eliminated, which may further suggest that regulation of peripheral nesfatin-1 secretion is mediated by an intrinsic (likely enteric) nervous mechanism. A more recent study found increased plasma levels of nesfatin-1 in cancer patients, but it is not clear whether it was of central of peripheral origin [29]. 

Another interesting property of peripheral nesfatin-1 is its beneficial effects during the course of several intestinal disorders. In the small intestine of neonatal rats with induced necrotizing enterocolitis (NEC), intraperitoneal injection of nesfatin-1 significantly reduced macroscopic and clinical scores and reversed the negative effect of oxidative stress by restoring and maintaining the balance between pro-oxidant and antioxidant mechanisms. Furthermore, the therapeutic anti-inflammatory and anti-apoptotic effects of nesfatin-1 observed in NEC-induced intestinal damage remained under the control of the afferent neurons and were linked to the protein’s ability to modulate the composition of the gut microbiota and the structure of the intestinal barrier [30]. It should be noted that previous research revealed an analogous role of nesfatin-1 as a factor balancing oxidative status in a rat small intestine model of acute mesenteric ischemia [31,32].

**Figure 2:**
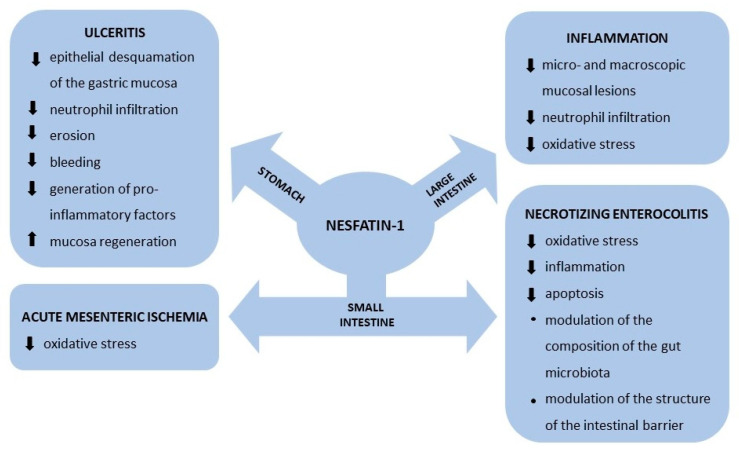
The effect of nesfatin-1 in selected GIT disorders.

In rats with acetic acid-induced inflammation of the large intestine, treatment with nesfatin-1 reduced micro- and macroscopic mucosal lesions by inhibiting neutrophil infiltration and suppressing free radical formation. As these effects were prevented by antagonists of both oxytocin and ghrelin receptors, the authors suggested a mechanism of nesfatin-1 anti-inflammatory signaling through both G-protein-coupled receptors [33] (for summary see Figure 2).

## 5. Conclusions

Although discovered over 15 years ago, the precise roles of peripheral nesfatin-1 in the regulation of the GIT are far from being fully understood. The identification and cloning of nesfatin-1 receptors would unquestionably be a milestone in further functional studies of the activity of peripheral nesfatin-1. Moreover, as some of the morphological reports are contradictory, knowledge of the level of nesfatin-1 expression in the gastrointestinal tract organs of various animal species requires systematization. The role of the peptide as a potential neurotransmitter or neuromodulator in the ENS is essentially unknown and has not been adequately explored. Although as yet there is no direct evidence that peripheral nesfatin-1 influences metabolic functions by interacting with the gut microbiota, this type of activity has been noted in the case of other food-intake controlling regulatory peptides [34]. Changes in glucose metabolism are generally regarded as one of the most important results of interaction between gut peptides and the gut microbiota, which makes nesfatin-1 an interesting subject molecule in research using animal models of diabetes. Due to the beneficial anti-inflammatory and anti-apoptotic properties of peripheral nesfatin-1, it is a potentially promising drug for treatment of gastrointestinal disorders.

## Data Availability

Data are available to be shared at any request.
